# Enhanced Solid Electrolyte Interphase Layer in Li-Ion Batteries with Fluoroethylene Carbonate Additives Evidenced by Liquid-Phase Transmission Electron Microscopy

**DOI:** 10.1021/acsnano.5c01460

**Published:** 2025-05-16

**Authors:** Walid Dachraoui, Ruben-Simon Kühnel, Nico Kummer, Corsin Battaglia, Rolf Erni

**Affiliations:** † Electron Microscopy Center, 28501 Empa—Swiss Federal Laboratories for Materials Science and Technology, Überlandstrasse 129, Dübendorf 8600, Switzerland; ‡ Materials for Energy Conversion, Empa—Swiss Federal Laboratories for Materials Science and Technology, Überlandstrasse 129, Dübendorf 8600, Switzerland; § Transport at Nanoscale Interfaces Laboratory—Swiss Federal Laboratories for Materials Science and Technology, Überlandstrasse 129, Dübendorf 8600, Switzerland; ∥ Department of Information Technology and Electrical Engineering, ETH Zürich, Gloriastrasse 35, Zürich 8092, Switzerland; ⊥ Institute of Materials, School of Engineering, EPFL, Station 15, Lausanne 1015, Switzerland; # Department of Materials, ETH Zürich, Wolfgang-Pauli-Strasse 10, Zürich 8049, Switzerland

**Keywords:** operando STEM, SEI, Li dendrites, FEC, electrolyte additives, Li-ion batteries, electrochemical liquid cell STEM

## Abstract

The solid electrolyte interphase (SEI) layer is essential for battery performance and safety due to its electron insulation and Li-ion conduction. However, issues such as ongoing electrolyte decomposition and Li dendrite growth often arise. The most common strategy for improving the SEI is using electrolyte additives. However, the growth mechanism of the SEI with additives remains unclear. In this study, we use operando electrochemical liquid cell scanning transmission electron microscopy (ec-LC-STEM) to monitor in real time the nanoscale processes at the electrode–electrolyte interface during battery operation. We investigate how the additive fluoroethylene carbonate (FEC) influences the formation and properties of the SEI, as well as the growth and dissolution of Li dendrites. Our study shows that FEC decomposes early, allowing the nucleation and growth of LiF nanoparticles (NPs) that create a dense, uniform, and thin SEI layer. Interestingly, our analysis reveals that these NPs have structural defects that could influence ionic and electronic conductivity. The real-time observations show that the FEC-based SEI facilitates the formation of dense and short Li metals, whereas the FEC-free SEI leads to the growth of long Li whiskers with thinner roots than tips. This structural difference influences their dissolution mechanism: in FEC-rich electrolytes, the strong contact between Li metal and the electrode ensures complete dissolution, while in FEC-free electrolytes, partial dissolution occurs, leaving behind inactive Li metal. These findings emphasize the crucial role of additives in shaping the growth mechanism and the local structure of the SEI, thereby regulating the growth and dissolution of Li metal.

## Graphical abstract

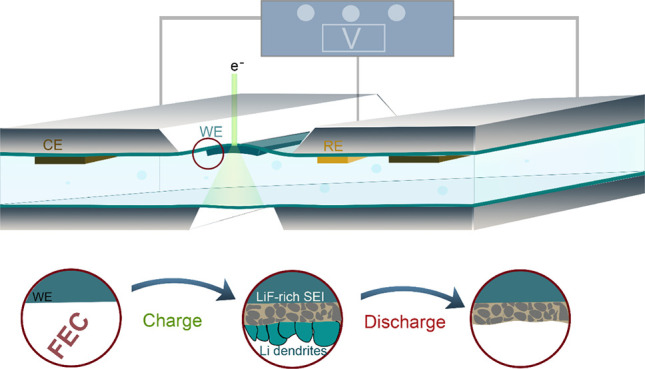

## Introduction

The longevity of Li-ion batteries hinges on the stability of the SEI throughout their cycles.^1–3^ During charging process, the electrolyte is consistently subjected to potentials beyond its stability range.^4^ This leads to its decomposition, causing the formation of the SEI at the anode–electrolyte interface.^5^ Particularly during the first cycle, the electrode surface is still exposed, making it highly susceptible to electrolyte decomposition. This process occurs at an elevated rate, producing numerous irreversible byproducts that consume Li from the reservoir. As a result, the system experiences significant capacity losses and low Coulombic efficiency.^6^ The SEI ideally serving as a protective barrier that suppresses further electrolyte degradation. The structure and functionality of this SEI depend on the chemical nature of the decomposition products and the specific electrode material.^7–9^ The formation of a thick SEI introduces resistance to Li^+^ intercalation kinetics, resulting in an elevation of the cell temperature during fast charging and accelerating the degradation of the cell.^10–12^ This self-accelerating process gradually depletes the electrolyte and reduces the amount of electrochemically active Li, leading to irreversible capacity loss in the cell. Furthermore, the nonprotective nature of the SEI layer can facilitate Li plating, which poses significant safety concerns.^13^

To mitigate the instability of the SEI and the growth of Li dendrites, numerous strategies have been proposed, including surface coating of electrodes and the incorporation of electrolyte additives.^14–16^ The formation of the SEI layer in the presence of electrolyte additives is based on the fundamental principle that additive molecules reduce at a higher potential than solvent and salt molecules during cycling, resulting in the creation of a stable SEI film. This effectively prevents the decomposition of electrolyte solvents and salt, enhancing the performance of Li batteries.^17–19^ Thus, electrolyte additives primarily modify the SEI layer by promoting the formation of a uniform SEI film with improved mechanical and structural properties.^20^ Consequently, batteries containing such additives exhibit prolonged cycling stability. The optimized SEI film facilitates Li-ion transport paths, diminishes the formation of Li dendrites, and thus improves the overall performance of the cell.^21^ One of the most important and widely used additives is FEC, a well-known additive extensively explored in rechargeable batteries, particularly for regulating the passivation layer of the Li anode in Li-metal batteries.^22,23^ This compound exhibits a higher reduction potential (1.1–1.4 V vs Li^+^/Li) compared to other carbonate solvents, like ethylene carbonate (0.7 V vs Li^+^/Li).^24,25^ Furthermore, the SEI formed with FEC is thermally more stable, maintaining stability up to around 200 °C, while traditional SEIs typically break down at approximately 153 °C.^26^ There are studies clearly showing that FEC benefits both Li- and Na-ion batteries.^27–34^ However, there is no clear understanding of how FEC improves cycling stability in LIBs and there is scarce information on the actual influence of FEC on the composition, structure, and formation mechanism of the SEI layer, which prevents the development of effective additives. Thus, understanding how the SEI forms in FEC-rich electrolytes and how this additive contributes to the formation of a thin and stable SEI layer is crucial. Moreover, comprehending the impact of FEC-derived SEI on the plating and dissolution of Li metal could lead to advancements in electrolyte design for achieving a stable interface. Achieving this understanding necessitates a technique with high spatial and temporal resolution to investigate these phenomena in real-time during battery operation while protecting the decomposition products for characterizations.

Over the past decade, ec-LC-STEM incorporating electron-transparent silicon nitride (Si_*x*_N_*y*_) windows has become a prominent method for studying phenomena in Li-ion batteries.^35–44^ In this study, we utilize ec-LC-STEM to investigate the SEI formation. We use a standard electrolyte consisting of 1 M LiPF_6_ in ethylene carbonate/ethyl methyl carbonate (EC/EMC (3:7 vol.)), without and with (10 wt %) FEC. Our real-time observations reveal that FEC enables the growth of a LiF-rich SEI layer. This occurs via the nucleation and growth of LiF-based NPs forming a thinner and denser inorganic layer than in the FEC-free electrolyte. The growth of this layer is followed by the decomposition of solvent molecules to form an organic film-like layer that serves as glue for the NPs. More importantly, our study shed light on the exact structure of the SEI layer and its effect on the Li dendrites formation, wherein the presence of FEC, the deposited Li metal is more compact and Li dendrites are shorter than those formed in additive-free electrolyte. This structural characteristic allows for the complete dissolution of the dendrites formed in the FEC-based electrolyte, in contrast to the pure electrolyte, where incomplete dissolution of the Li dendrites occurs, generating dead Li.

## Result and Discussion

### Formation of SEI Layer: 0% FEC vs 10% FEC

We focus on imaging the solid–liquid interface during the SEI growth and Li metal plating with high spatiotemporal resolution. The growth of the SEI was investigated in real time using cyclic voltammetry (CV) within an ec-LC-STEM. The structural changes were monitored through annular dark-field scanning transmission electron microscopy (ADF-STEM) imaging as the applied potential was varied. Schematics illustration of the electrochemical microchip are shown in . Figure S1a shows the three-electrode configuration consisting of a glassy carbon (GC) as working electrode (WE) and Pt serving as both the counter electrode (CE) and reference electrode (RE) (hereafter referred to as Pt). The potential of 0 V in this configuration corresponds to approximately +3 V versus Li/Li^+^, calibrated using a solution of 1 mol/L LiPF_6_ in EC/EMC (3:7 by volume) with 10 mmol/L ferrocene (Fe­(C_5_H_5_)_2_).^5^ Figure 1a presents a typical atomic force microscopy (AFM) image depicting the GC WE positioned on top of the Si_3_N_4_ membrane. The image reveals that the edge of the GC is sharp which facilitates the study of the growth and the morphology of the SEI layer. GC was selected as a model system due to its inertness toward Li intercalation, its stability during repeated cycling, and its composition, which reflects the carbon black additives commonly used in commercial batteries. Its low electron scattering cross-section makes GC an ideal low-contrast electrode material for S/TEM imaging.^45^ Additionally, GC consists predominantly of electrically conductive sp^46^ hybridized carbon, similar to graphite but does not allow significant alkali ion intercalation. As a result, any contrast variations observed during imaging can be directly attributed to SEI formation, Li electrodeposition, or changes in material density, rather than to electrode expansion caused by Li-ion insertion.^47^ The common electrolyte, LiPF_6_ in EC/EMC (3:7 by volume) was chosen as our model system, both without (0%) and with (10 wt %) FEC additive. The FEC additive is known to promote the formation of an F-rich interphase in such carbonate-based electrolytes.^48^ All ec-LC-STEM experiments were performed at low dose rates to minimize beam damage by radiolysis.^5^ A control experiment was conducted to monitor the effects of a comparable dose on the electrolyte, showing no observable breakdown products from the beam (see ).

**Figure 1:**
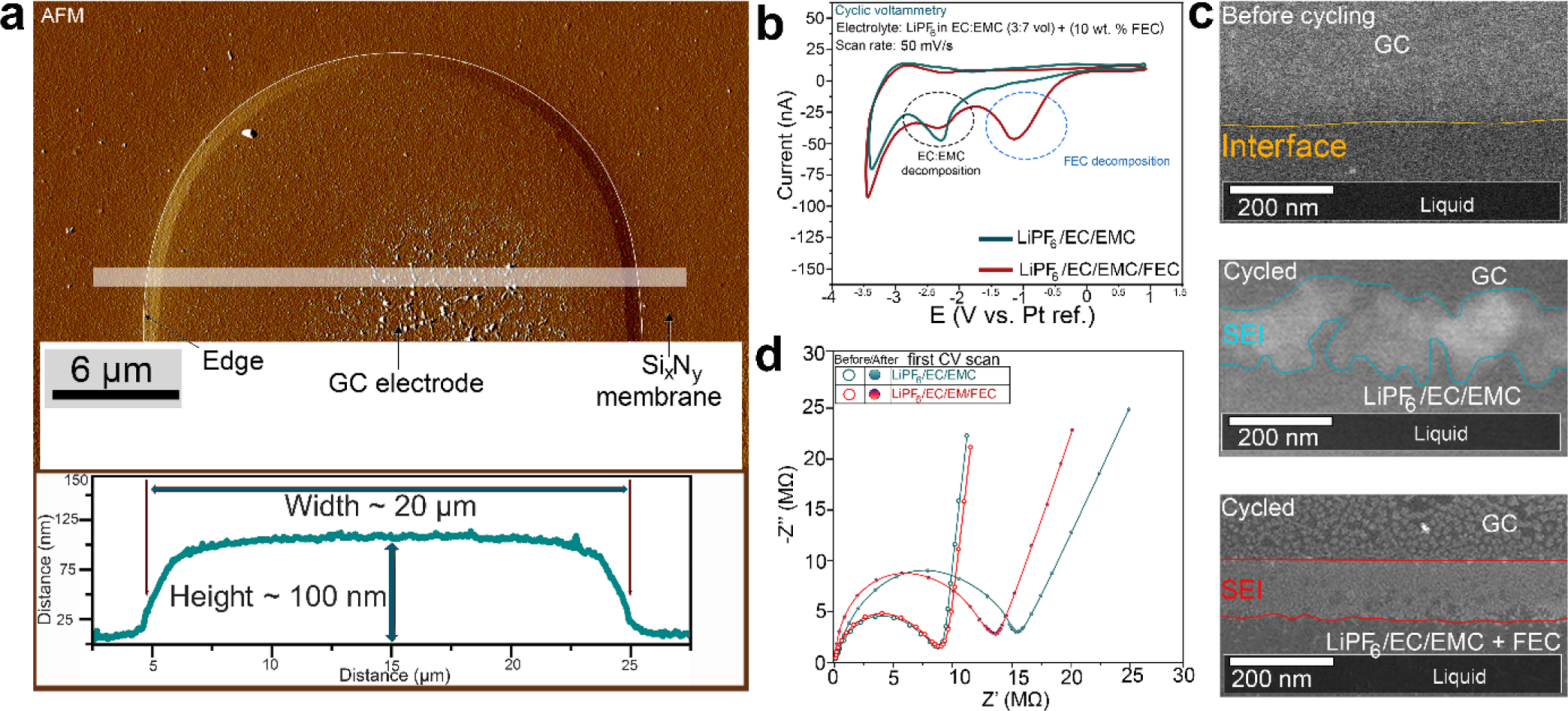
SEI formation of a GC electrode without and with 10% FEC additive. (a) AFM micrograph of the GC electrode. (b) Two typical CV curves of the micro battery cycled in the additive-free electrolyte and with 10% FEC. (c) ADF-STEM images of the GC edge before and after cycling with both electrolytes. (d) EIS of the operando cell with FEC-free electrolyte and 10% FEC electrolyte before and after cycling.

Typical CV curves of the electrochemical cell with the electrolyte, both with and without FEC, measured at 50 mV/s, are shown in Figure 1c. The voltammogram for the cell with FEC-free electrolyte is represented in green and shows a single peak around −2 V vs Pt, corresponding to the decomposition of the electrolyte.^5^ The voltammogram for the cell containing FEC is represented in red and exhibits two peaks: one around −1 V vs Pt, corresponding to the decomposition of FEC, and a second smaller peak at −2 V vs Pt, corresponding to the decomposition of EC/EMC. Figure 1c displays ADF-STEM images of the interface between the GC and the electrolyte, captured before cycling (top), after cycling for 0% FEC (middle), and after cycling for 10% FEC (bottom).This observation aligns with previous studies.^24^ It is well-known that FEC reduction takes place at a more positive potential compared to EC and EMC reduction.^24^ In comparison to the SEI layer formed without FEC, which exhibits an inhomogeneous morphology, the SEI layer formed in the FEC-rich electrolyte is thinner and more compact (Figure 1c).

Electrochemical impedance spectroscopy (EIS) measurements were conducted to determine the conductivity at the anode–electrolyte interface before and after cycling for both electrolytes. Nyquist plots for the cells with 0% and 10% FEC, include semicircles in the high-frequency region (Figure 1d), which is ascribed to Li^+^ migration through the SEI on the negative electrode surface.^49^ Before cycling, the interface resistance for both electrolytes is approximately 8 MΩ (the resistance is so high due to the low surface area of the GC electrode). However, after cycling, there is a notable increase in SEI resistivity for both cells. This increase differs between the 0% and 10% FEC electrolytes, where the SEI of 10% FEC shows a lower resistivity (∼13 MΩ) compared to the SEI of 0% FEC (∼16 MΩ). This suggests that the SEI layer with 10% FEC has a higher Li^+^ conductivity compared to the FEC-free SEI. After five cycles, the 10% FEC cell exhibited significantly lower SEI resistance than the 0% FEC cell (). Consequently, the SEI formed in the FEC electrolyte is more stable and conductive, resulting in higher Li^+^ conductivity. This confirm that FEC in addition to controlling the morphology can influence the composition of the SEI film produced on the surface of the GC anode. The equivalent circuit used to fit the EIS curves is shown in . Here, ADF-STEM imaging combined with EIS demonstrates that both the morphology and the ionic conductivity are improved with the addition of FEC. In order to understand the different properties, the growth mechanism and the structure of the FEC-based SEI need to be inspected in detail.

### Decomposition of FEC Additive and Mechanism of the Corresponding SEI Growth

We investigated the initial stage of SEI growth in both electrolytes using in ex-situ electrochemical LC-STEM. Figure 2a displays a CV curve obtained from the microcell filled with the FEC-free electrolyte at a scan rate of 50 mV/s. The highlighted peaks from 1 V to −3.5 V vs Pt correspond to the charging process. Figure 2b shows the current vs time curve during the charging process. Two redox peaks emerge during the first lithiation. The first peak, located at approximately at −1 V vs Pt and highlighted in blue in Figure 2a, corresponds to the decomposition of FEC.^20^ The second peak appears around −2.5 V vs Pt, as highlighted in gray in Figure 2a. We imaged the edge of the GC electrode during this process. Figure 2c displays a series of sequential ADF-STEM images extracted from , depicting the early stages of the SEI layer growth. Based on the CV curve in Figure 2a and the current vs time curve in Figure 2b, the decomposition of FEC occurs within the time frame of 0 to 55 s. The time point *t* = 0 s marks the start of the CV cycle, initiated at 1 V vs Pt with a scan rate of 50 mV/s. At this initial stage (*t* = 0 s), the GC–electrolyte interface outlined by an orange dashed line in Figure 2c, appears smooth and well-defined. After 10 s of cycling corresponding to the onset of the first peak of decomposition (blue), small clusters begin to form at the edge of the electrode indicated by yellow arrows in Figure 2c. These clusters observable by their bright contrast, can be attributed to the decomposition products of the FEC additive and form a loose layer highlighted by a red dashed line. Between *t* = 30 s and *t* = 50 s, the growth of the clusters continues, resulting in an increase in particle size and the formation of a thin bright layer at the edge of the GC electrode, with an approximate thickness of 150 nm. The enlargements in Figure 2c (false color) at *t* = 10 s and *t* = 50 s, corresponding to the regions highlighted by the white squares, clearly show that the layer is primarily composed of nanoclusters.

**Figure 2:**
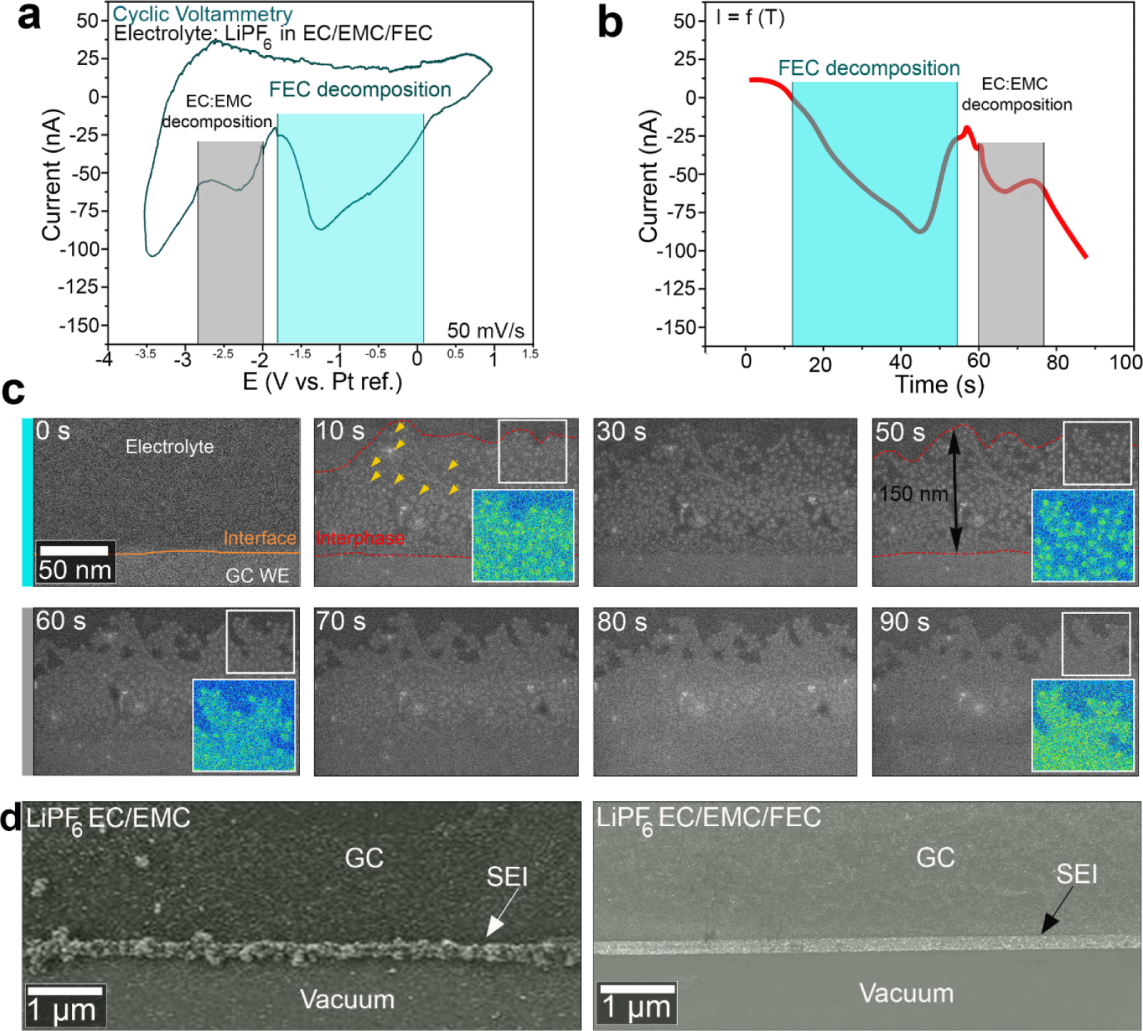
Operando studies of the nucleation and growth of the SEI on a GC electrode in the electrolyte with 10% FEC. (a) Cyclic voltammetry with a scan rate 50 mV/s. (b) Change in the current versus time during the charge process. (c) Time-lapse series of ADF-STEM images showing the SEI growth during the charging process (frames from ). (d) SEM images of the SEI formed in the electrolyte without (left) and with (right) FEC.

From *t* = 50 to 60 s the growth of the NPs slows down. Nonetheless, the layer’s image contrast kept evolving, when at *t* = 60 s an evident change in the contrast of interparticle regions is visible (also see for more details). By the end of the charging process, at around *t* = 90 s, the layer built by individual NPs became completely enveloped by a matrix layer covering the NPs, which appears to act as a glue. This secondary film-like layer likely results from the decomposition of the carbonate solvents EC and EMC. A clear reaction pathway of the SEI growth from the FEC-based electrolyte can be outlined: *growth of nanoparticles* during the FEC decomposition followed by the growth of a *thin organic layer* during the decomposition of solvent molecules.

In addition to the SEI layer, tiny bubbles appeared (see Figure S5a), indicating the generation of gaseous products. To confirm that the SEI and the bubbles were generated by electrochemical cycling rather than by e-beam irradiation, a control experiment was conducted. The results demonstrated that, in the absence of applied CV neither the bubbles nor the formation of a layer on the GC electrode were observed (see ). In a separate experiment, the LC was subjected to the same CV sweep, this time performed outside of the TEM. Imaging conducted at the end of the sweep revealed the formation of a similar SEI layer (Figure S5b). Thus, NP growth and SEI formation occur exclusively under CV conditions in the electrochemical LC, ruling out a beam-induced effect. Although electron-beam effects are complex and challenging to quantify, our control experiments confirm that beam-induced effects are negligible in our studies. Therefore, the formation of bubbles is due to gaseous species produced by electrochemical LiPF_6_, EC and/or EMC decomposition, likely CO, CO_2_ and/or PF_5_, and FEC decomposition, likely CO_2_ and H_2_.^45,49,50^ The highly confined volume of the LC promotes the coalescence of the small gas bubbles produced across the electrode, leading to the formation of individual bigger bubbles. (Figure S5c). Bright dots can also be observed in the liquid area surrounding the GC electrode. These are likely due to the detachment of NPs from the large surface of the GC electrode or the partial dissolution of organic compounds in the electrolyte (Figure S5b).

In the FEC-free electrolyte, the growth pathway and final morphology of the SEI layer differ from the FEC-based electrolyte. In the FEC-free case, we observed the SEI formation, as shown in , which presents time-lapse ADF-STEM images of the SEI growth. In this case, the growth is sudden, with a block of material deposited at the edge of the GC WE. At *t* = 90 s, an SEI layer is observed. Unlike the homogeneous SEI layer formed in FEC-based electrolyte, this SEI has a nonuniform morphology with a certain degree of porosity (indicated by yellow arrows in ).

In order to visualize the overall morphology of the SEI layers formed without and with FEC, the top microchips of both electrochemical cells were studied after 5 cycles using SEM. Figure 2d shows two typical SEM images depicting the edge of the GC WE after five cycles in without (left) and with FEC (right). These SEM images clearly show a difference in morphology and thickness, where the presence of FEC enables the formation of a thinner and sharper SEI layer compared to the FEC-free electrolyte.

Our real-time operando ec-LC STEM analysis reveals that SEI growth with FEC initiates with the nucleation and expansion of a layer of NPs, followed by the formation of an organic layer interconnecting the NP agglomerate layer. To gain a deeper understanding of the growth mechanism of the NP layer, we evaluated the size evolution of NPs during the charging process. Figure 3a presents a time-lapse ADF-STEM image (in temperature contrast) of a representative NP captured from the edge of the GC electrode (extracted from ). The edge of the NP is outlined with a white dashed line, with arrows indicating its expansion over time during the charging process. By examining the projected surface area of the NPs, we tracked the surface evolution of three NPs (designated P1, P2, and P3 in ) over time, as shown in Figure 3b. The data indicate a consistent surface evolution trend among all NPs: they exhibit rapid growth from *t* = 10 s to *t* = 40 s, followed by a deceleration in growth kinetics until *t* = 50 s, at which point growth ceases. We also monitored the distance between two NPs (Figure 3a-bottom extracted from ) over the same time interval, with the corresponding curve shown in pink in Figure 3b. The distance between the NPs decreases rapidly from 10 to 40 s, reaching 20 nm, after which the rate of change slows down and stabilizes after 50 s. This behavior aligns with the NPs size growth, indicating that the increase in NPs size is the primary factor driving the development of the SEI layer into a continuous, compact structure. Figure 3c presents a schematic illustration depicting the nucleation and evolution of NPs through FEC decomposition. In this process, a continuous compact layer is formed by the agglomeration of the growing NPs, which is followed by the decomposition of EC, which generates an organic layer that acts as glue for the NPs. These observations provide unique insights into the precise mechanism of the SEI formation at the interface between the GC electrode and the electrolyte with FEC. Further insights can be gained by examining the chemical composition and structure of the FEC decomposition products.

**Figure 3:**
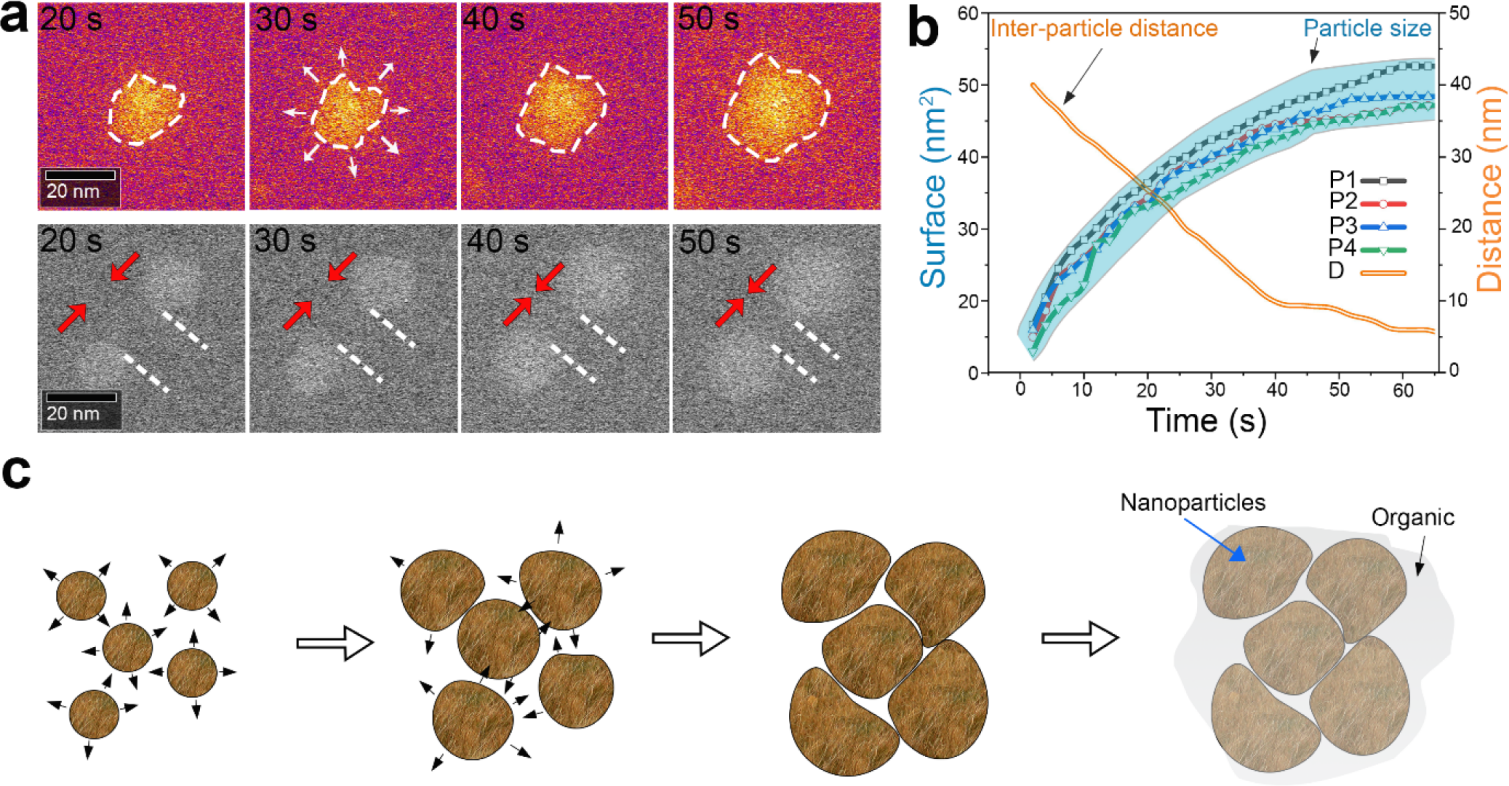
Nucleation and growth of inorganic nanoparticles by decomposition of FEC. (a) Time-lapse ADF-STEM images showing the growth of an individual nanoparticle (top) and two neighboring nanoparticles (bottom) during the charging process. (b) Plots illustrating the evolution of surface area over time for four different particles and the interparticle distance versus time during the charging process. (c) Schematic illustration of the nanoparticle evolution and the formation of a film-like layer.

### Atomic Structure and Chemistry of FEC Decomposition Products

The thick liquid medium of the cell with the Si_3_N_4_ membranes hindered our ability to achieve sufficient spatial resolution to study the atomic structure of the NPs generated during FEC decomposition. In order to study in depth the compounds forming the SEI layers of both cells (i.e., with and without FEC), ex situ high-resolution (HR)-ADF-STEM analysis was performed. This was done after expelling the liquid electrolyte with argon and gently cleaning the microchip inside a glovebox to remove any organic residues (additional details are provided in the Experimental Details section). Figure S8a presents a typical ADF-STEM image of the SEI formed without FEC. By analyzing the corresponding fast Fourier transforms (FFT), we determined the atomic structure of the crystalline phases. Figure S8b shows three HR-ADF-STEM images collected from three distinct regions, marked by blue, red, and green squares in Figure S8a. Three types of lattice fringes were detected that could be related to lithium fluoride (LiF), lithium oxide (Li_2_O), and lithium carbonate (Li_2_CO_3_), respectively. This indicates that the inorganic layer of the SEI consists of various compounds distributed randomly. Indeed, this confirms the coexistence of the inorganic and organic compounds forming a mosaic SEI layer.^51^ The predominance of these inorganic compounds suggests that the species formed at the initial stages are indeed mainly inorganic.^5^ However, reveals that the formed SEI layer exhibits a porous structure, likely due to the presence of organic compounds enriched at the outer layer, as highlighted by black arrows.

An equivalent study was conducted for the SEI formed with the FEC-rich electrolyte. Above we showed that FEC decomposition generated a nanoparticle-rich SEI layer, making it worthwhile to study the atomic structure and the chemical composition of these NPs. Figure 4a presents an overview ADF-STEM image of the formed SEI layer in the FEC case, where bright dots are visibly embedded in a less bright layer. One can see that the number and the concentration of particles are less than those in liquid mode. We believe that this is due to the cleaning process. Figure 4b shows a single NP imaged using HR-ADF-STEM, which possesses an elliptical shape and is composed of ultrasmall primary particles. This is shown in the magnification of Figure 4b, where a side of a primary particle is highlighted with dashed red lines. The secondary particles measure approximately a few nm to 60 nm, while the primary particles range from 1 to 3 nm. The FFT pattern shown on the right side of Figure 4b can be indexed with two different phases: LiF (yellow) and Li_2_CO_3_ (green). This indicates that the secondary particles are formed by an agglomeration of LiF and Li_2_CO_3_ nanodomains, which is in agreement with the study conducted by Lu and coworkers.^2,4^ Small spots also appear in the FFT pattern and can be indexed to the Li_2_O phase (indicated by blue arrows in the FFT in Figure 4c). To quantify and identify the dominant compound in the NP, we selected the rings in the FFT pattern corresponding to Li_2_CO_3_ and LiF, then calculated the corresponding virtual ADF-STEM images. Figure 4c illustrates a section of the primary NP, highlighting the Li_2_CO_3_ domains in green and the LiF domains in yellow based on the inverted FFT calculations. LiF appears to be the dominant phase, while Li_2_CO_3_ is present as a few small, randomly distributed domains. However, when selecting the spots corresponding to the Li_2_O phase, no fringes are visible in the corresponding image, indicating that Li_2_O is present in very small amounts within a phase dominated by LiF and Li_2_CO_3_. Figure 4d shows three typical atomic resolution ADF-STEM images from the yellow region with the corresponding FFT patterns. The indexation of the FFT patterns and the *d*-spacings support the presence of the LiF phase. Moreover, we aimed to shed light on the atomic structure of the connections between the secondary particles. Figure 4e presents three typical HR-ADF-STEM images of the grain boundaries (GBs) between different domains. Various defects are visible at the boundaries and at the core of the NPs, including twin boundaries (TBs), stacking faults, and structural distortions. These defects can be attributed to the different orientations of the LiF grains and the structural differences between Li_2_CO_3_ and LiF.

**Figure 4:**
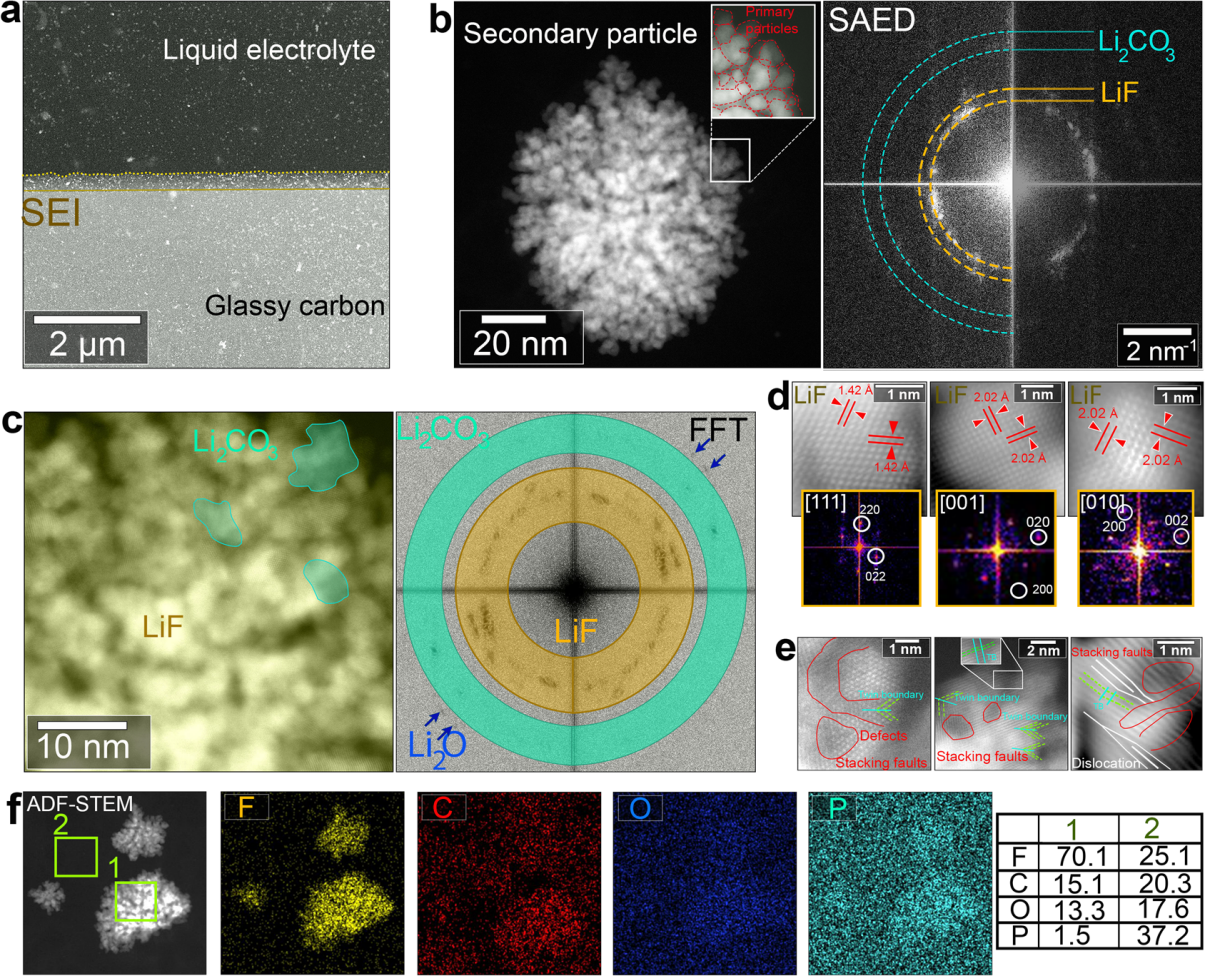
Nucleation and growth of inorganic nanoparticles formed by the decomposition of FEC. (a) Overview ADF-STEM image of the SEI layer formed in the FEC-containing electrolyte. (b) HR-ADF-STEM image of a typical NP with its corresponding FFT pattern. (c) HR-ADF-STEM image of a nanoparticle where LiF and Li_2_CO_3_ phases are highlighted based on virtual FFT pattern (left). (d) HR-ADF-STEM images of three different regions with their corresponding FFT patterns. (e) HR-ADF-STEM image showing defects and grain boundaries. (f) EDS mapping of a region containing three NPs.

We further performed energy-dispersive X-ray spectroscopy (EDS) mapping to analyze the local distribution of elements in a region containing three NPs. The EDS measurements were carried out ex situ on the same sample used for HR-STEM analysis. The EDS maps shown in Figure 4f reveal information about the distribution of the elements, indicating an inhomogeneous presence of C, O, F, and P. No signs of electron-beam-induced damage are observed. The combination of ADF-STEM and EDS mapping indicates that the NPs are dominated by fluorine, which is in agreement with the HR-STEM results and can be attributed to the LiF compound. The elemental composition was examined through EDS point analysis at specific locations within the SEI layer (points 1 and 2 in Figure 4f), with the corresponding spectra presented in . The NP region (region 1) shows a significant concentration of F contrary to region 2, which shows significant amounts of C, O, and P with little F. This can be related to organic compounds (e.g., ROLi, ROCOOLi, and RCOOLi) being dominant in region 2. Both regions show a notable concentration of carbon, which can be attributed to their proximity to the glassy carbon surface. Thus, a combination of the HR-STEM and EDS analysis allows to support the model of a compact layer of inorganic NPs, mainly rich in LiF, embedded in an organic layer. This is in good agreement with previous reports.^52^

### Li Dendrite Growth and Dissolution in Electrolytes with FEC vs without FEC

Real-time investigations of Li plating and stripping was conducted using CV with both cells extending the cell voltage to lower potentials (). The structural dynamics were captured using ADF-STEM imaging while varying the applied potential. Regions appearing with the darkest contrast are attributed to lithium metal, identifiable by its significantly lower density relative to the electrolyte.^53^ To enhance the visibility of the Li dendrites and dead Li metal, the contrast was inverted as shown in the magnified inset in Figure 5a,b (green rectangles). In the absence of FEC in the electrolyte, Li deposition begins with nucleation at the edge of the GC working electrode, followed by rapid growth in the form of whiskers through a root-growth mechanism (more details in ). Figure 5a shows an overview of the GC edge after charging where long whiskers are formed, as highlighted by blue dashed lines in the magnified inset in Figure 5a. These whiskers have tips thicker than the root. After the discharge process grains of dead Li disconnected from the GC electrode are formed and clearly visible as highlighted with red dashed lines in the magnified inset of Figure 5b. Figure 5c shows a time-lapse ADF-STEM images (inverted contrast) depicting the dissolution process of Li dendrites during discharge. The underlying process is described in one of our earlier works.^54^

**Figure 5:**
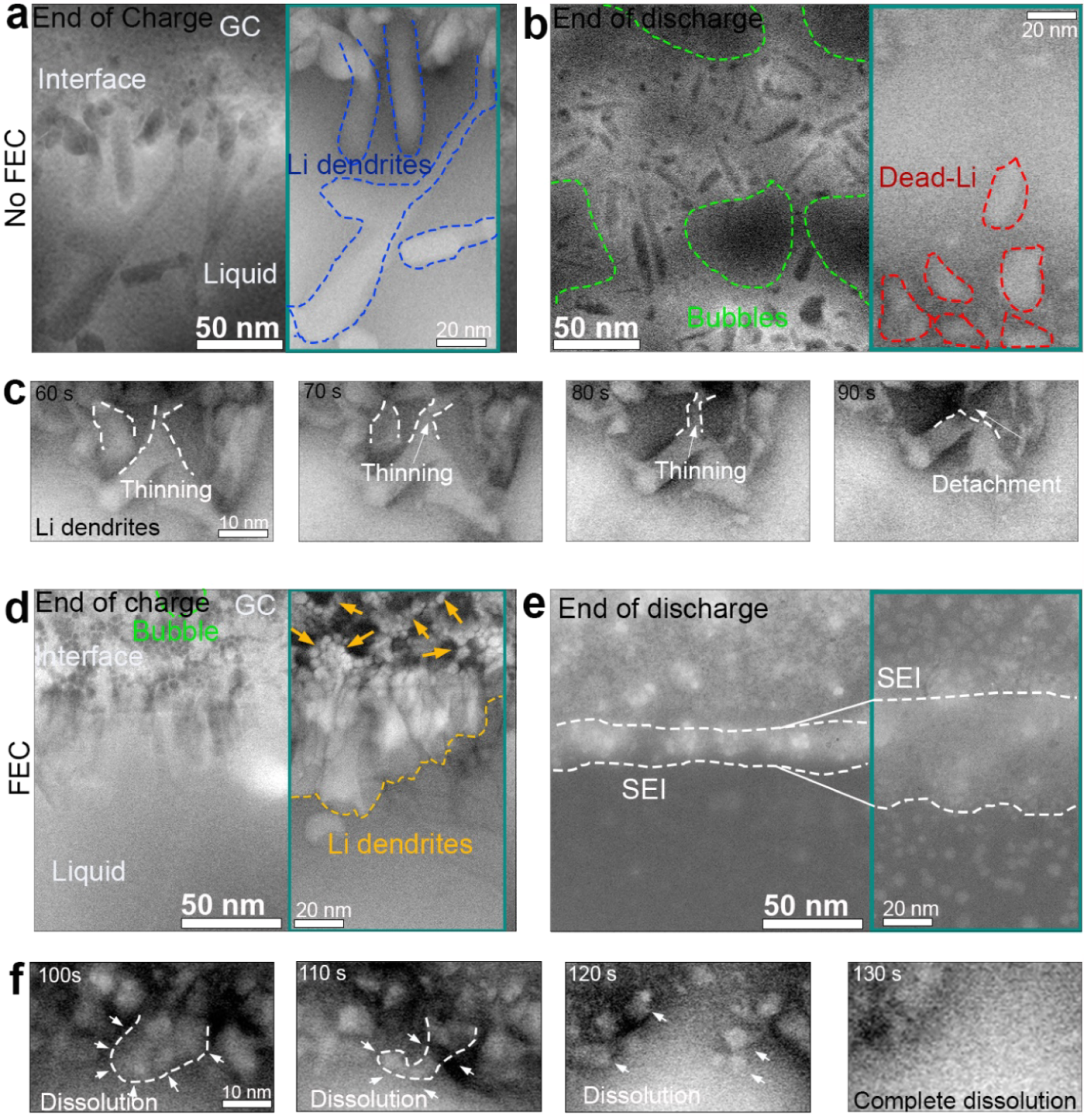
Li dendrite growth and dissolution with and without FEC. (a) ADF-STEM image of Li dendrites grown in electrolyte without FEC. (b) ADF-STEM image of dead Li formed in electrolyte without FEC. (c) Time-lapse ADF-STEM images showing dissolution of Li dendrites formed in electrolyte without FEC. (d) ADF-STEM image of Li dendrites grown in the electrolyte with FEC. (e) ADF-STEM image of dead Li formed in the electrolyte with FEC. (f) Time-lapse ADF-STEM showing dissolution of Li dendrites formed in electrolyte with FEC.

Identical experiments were carried out to study the Li plating and stripping processes in an FEC-rich environment. The morphology of the plated Li is different from the one of Li formed in the FEC-free electrolyte, where the FEC-based SEI allows forming denser and shorter micron-sized grains with higher connectivity. In the magnified view in Figure 5d (green rectangle), it is shown that the plated Li pieces are effectively connected to each other. and the roots are located at the empty space (grain boundaries) between the inorganic NPs of the LiF-rich SEI layer (highlighted with yellow arrows). The uniform and relatively flat morphology of the Li deposit facilitates stable electrical contact with the WE throughout stripping. This enables a consistent stripping process that initiates at the tips and proceeds toward the base, as shown in Figure 5f, which allows complete dissolution of the deposited Li (more details in ). Hence, while the specific morphology of the Li dendrites in the FEC-free electrolyte leads to the formation of dead Li, no dead Li is observed in the FEC-containing system.

Our in situ ec-LC STEM experiments revealed a marked difference in SEI growth behavior between the two electrolytes­(i.e., LiPF_6_ in EC/EMC without and with 10 wt % FEC). A schematic summary of our in situ observations, namely the SEI growth as well as the early stages of Li plating and its dissolution, without and with FEC-containing electrolytes is given in Figure 6. Upon charging, the FEC-free electrolyte decomposes and the SEI film is instantly deposited on the WE. This generates a layer of a mixture of organic and inorganic species (Figure 6-No FEC). The formation of the SEI layer in this electrolyte containing LiPF_6_, involves the decomposition of EC and EMC. This process yields a variety of inorganic and organic species.^5^ Among the inorganic components we find LiF, from the decomposition of LiPF_6_, and Li_2_CO_3_, resulting from the breakdown of EC and EMC.^49^ Concurrently, the decomposition of EC and EMC generates organic species such as alkyl carbonates and ethylene (more details in SI). This simultaneous formation of organic and inorganic species results in an inhomogeneous SEI layer with defects and pores that facilitate further contact between the electrolyte and the anode. However, in the presence of FEC, the growth mechanism of the SEI layer is completely different. Upon charging of the cell, FEC decomposes preferentially prior to the main electrolyte constituents due to its high reactivity.^24^ During the charge process, FEC undergoes reductive decomposition on the negative electrode surface, producing LiF. In this process, FEC reduction leads to the release of F^–^ ions that react with Li^+^ to form LiF (more details in Supporting Information). The decomposition of FEC may also occasionally produce Li_2_CO_3_, although less frequently observed compared to LiF. The main differences between the two SEIs are thus not of chemical but of structural nature, resulting from the specific formation processes. Here, we use GC due to its properties (outlined above), which facilitate a clearer interpretation of the TEM data. However, we believe that the process of FEC and the electrolyte decomposition could be universal across different anode materials, such as Li metal and graphite. Nevertheless, the deposition of the decomposition products may be influenced by the unique properties of each anode material, including surface roughness and defects.

**Figure 6:**
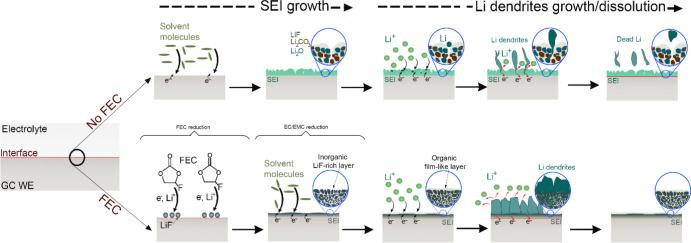
Schematic illustration of SEI growth in LiPF_6_ in EC/EMC (3:7 by volume), comparing conditions with and without FEC, and their effects on the growth and dissolution of Li dendrites.

Our in situ study complemented by specific ex situ analyses, revealed that during the charge process, the SEI growth initiates by nucleation of ultrasmall LiF NPs at the WE. This growth involves an initial sudden nanocluster formation followed by size expansion until the entire surface is covered. These LiF NPs appear to be secondary particles, consisting of ultrasmall primary particles rich in defects. The further agglomeration of these secondary particles allows the formation of a thin, dense LiF-based layer. Thereafter, other electrolyte components decompose, which allows the formation of an organic layer acting as glue for the initially formed inorganic LiF-based layer, by filling the space between the grains. The FEC is thus sacrified to form a thin inorganic layer primarily composed of LiF NPs. LiF is characterized by its ability to conduct ions on its surface while acting as an electronic insulator.^55^ This dual property means that this layer significantly limits the transfer of electrons to the solvent molecules of the electrolyte. Unlike organic solvents (i.e., here EC and EMC), which decompose through direct electron transfer processes, LiPF_6_ does not decompose in the same way.^56^ Thus, EMC/EC decompose first due to their more positive reduction potential and higher susceptibility to decompose with fewer electrons compared to LiPF_6_. Interestingly, the in situ observations here reveal that NPs first nucleate and then grow in both size and number, likely through atomic deposition. This process facilitates the formation of defects such as twin boundaries (TBs) and grain boundaries (GBs), which can significantly improve the properties of LiF. The TBs enhance ionic conductivity and mechanical strength by facilitating the migration of Li ions and impeding the movement of dislocations. On the other hand, the GBs offer additional ionic pathways, improve chemical reactivity, and help with impurity segregation. Together, these defects contribute to the formation of a stable and robust SEI layer, which supports uniform Li deposition. Indeed, our real-time imaging shows that without FEC, the formed SEI promotes the generation of long, dispersed whiskers with roots thinner than the tips. However, the FEC-derived SEI aids in generating short and dense Li dendrites that are interconnected. This is in agreement with the study conducted by Lin and coworkers, which shows that the introduction of FEC to solid polymer electrolytes promotes the formation of a robust SEI layer and enables the growth of Li dendrites with a dense and uniform morphology, in contrast to the porous and dendritic morphology generated without FEC.^33^

During the discharge process, our real-time observations show that without FEC, dead Li forms, whereas with FEC a complete dissolution of the Li dendrites is possible. The formation of dead Li follows the mechanism revealed in one of our earlier studies^54^ (Figure 6-NO FEC). In contrast, our high-resolution STEM imaging demonstrate due to its relatively flat and uniform morphology, the Li deposit largely retains electrical contact with the working electrode during the stripping process. This ensures a smooth stripping process from the tip to the base, preventing the formation of dead Li (Figure 6-FEC). Thus, The dissolution of Li metal is driven by two factors: its compact morphology and the etching of the surrounding SEI layer enabling direct contact between the Li metal tip and the electrolyte.^54^ This finding enhances our understanding of how electrolyte additives influence the structure and morphology of the SEI, which in turn affects Li plating and stripping, and eventually the cycling stability of the battery.

## Conclusions

Our real-time imaging of SEI growth and the dynamics of Li plating/stripping in an FEC-rich environment has highlighted the crucial role of this additive in enhancing the characteristics of the anode–electrolyte interface. The presence of LiF resulting from FEC decomposition, not only enhances the stability and robustness of the SEI layer but also minimizes the organic species resulting from the decomposition of the electrolyte solvents and salt. More importantly, the growth mechanism as well as the complex morphology and structure of the LiF NPs promote the formation of crystal defects within the SEI layer. The thin, compact and homogeneous SEI layer rich in defects influences the uniformity of Li metal deposition by encouraging the growth of short and dense Li dendrites, which help to prevent the formation of dead Li and internal short circuits. This study demonstrates the effectiveness of combining in situ electrochemical LC with STEM to elucidate the precise mechanisms of FEC-based SEI formation at the nanometer scale. Where it provides detailed insights into the structural and chemical properties of the SEI layer and its direct role in Li dendrites growth. Importantly, our research highlights the critical importance of well-designed electrolytes and emphasizes that in situ methods are essential for accurately observing the fundamental processes in such complex systems at the nanometer scale. As a perspective, it would be valuable to extend this study to investigate a wider range of anode materials and assess the properties of the LiF-rich SEI layer formed over Li metal or graphite-based anodes. While this study sheds light on the early stages of LiF-rich SEI formation on a GC anode, a broader exploration could provide deeper insights into how the decomposition of FEC, followed by electrolyte breakdown, is influenced by the characteristics of various anodes (e.g., Li metal and graphite-based). Such investigations could reveal how different electrode materials affect the formation, stability, and functionality of the SEI layer, as well as its influence on Li plating/stripping behavior. This knowledge would be essential for optimizing electrolyte design, ultimately enhancing the performance, safety, and longevity of lithium-based batteries.

## Methods

### Materials

Solution of lithium hexafluorophosphate in ethylene carbonate (EC) and ethyl methyl carbonate (EMC) with the composition 1 mol/L LiPF_6_ in EC/EMC = 3/7 (v/v) (99.9%). Electrolyte: 1 mol/L LiPF_6_ in EC/EMC = 3/7 (v/v) + 10% fluoroethylene carbonate (FEC). The electrolytes, bis­(cyclopentadienyl)­iron, di­(cyclopentadienyl)iron (Fe­(C_5_H_5_)_2_, ferrocene), and FEC were purchased from Solvionic. Acetone, methanol, and dimethyl carbonate (anhydrous, 99%) were purchased from Sigma-Aldrich. All chemicals were used without further purification. Microchips for the electrochemical LC were purchased from Protochips.

### Experimental Details

The experiments were conducted using an FEI Titan Themis 80-300 S/TEM, equipped with a probe Cs-corrector and operated at an accelerating voltage of 300 kV. ADF-STEM mode was primarily used to visualize the samples, offering atomic-number-sensitive contrast (Z-contrast) with intensity scaling approximately as Z^*n*^ (where *n* ranges between 1.6 and 1.8). SEM characterization was carried out on a Zeiss GeminiSEM 460, coupled with an Ultim Extreme EDS detector for elemental analysis. Complementary topographical analysis of the glassy carbon (GC) electrode was performed via AFM using a Bruker Icon 3 system in tapping mode. The scans were acquired with NuNano SCOUT 150 HAR RAu probes, characterized by a 150 kHz resonance frequency, an 18 N/m spring constant, and a nominal tip radius of 10 nm. The in situ TEM experiments were conducted using a Protochips Poseidon 510 liquid-cell holder and the electrochemical measurements were performed with a Gamry Instruments Reference 620 Potentiostat/Galvanostat/ZR.

The electrochemical LC is assembled using two microchips (E-chips). The top chip integrates three electrodes: a GC electrode serving as the WE, and Pt electrodes functioning as the reference and counter electrodes, respectively (see Figure S1a). The bottom microchip, referred to as the spacer chip, contains nanoscale spacers (see Figure S1b), approximately 150 nm in height in this study, which define the liquid layer thickness.

The preparation of the experimental setup involves several steps:

1. *Microchip cleaning:* both microchips are cleaned using a conventional solvent-based procedure, involving immersion in acetone for 4 min followed by isopropanol for 4 min. They are then gently dried with air.
2. *Cell assembly:* the cleaned E-chips are carefully aligned and stacked to avoid damaging the fragile silicon nitride membranes. Using O-rings, the chips are assembled into a sealed liquid cell mounted on a Protochips Poseidon holder.
3. *Seal testing:* the holder is first placed in a vacuum station to evaluate the integrity of the seal under high vacuum conditions.
4. *Holder insertion and setup connection:* the holder is transferred to the TEM, after which the potentiostat is connected to the electrical contacts, and the syringe pump containing the electrolyte is connected to the holder’s fluidic tubing (see ).
5. *Electrolyte injection:* the electrolyte is injected at a flow rate of 2.5 μL/min for 30 min, with the e-beam turned off during the injection.
6. *Calibration:* initially, we calibrated the system for 300 s and conducted open circuit measurements.
7. *Imaging conditions optimization:* although the use of an electron beam is essential for imaging in ec-LC TEM experiments, it can introduce undesired effects, particularly through radiolysis of the electrolyte; The interaction of the beam with the electrolyte may generate solvated electrons and free radicals, which can chemically reduce Li^+^ ions and potentially lead to Li precipitation within the electrolyte. To minimize such artifacts, we carefully optimized the imaging conditions by employing a moderate electron dose rate (∼0.5 e^–^/Å^2^·s). Under these conditions, no signs of Li precipitation were observed during imaging. This is evidenced in , which shows representative ADF-STEM images of GC in contact with the electrolyte (with and without FEC). Even after 10 min of continuous exposure, the absence of visible Li precipitation suggests that the chosen imaging parameters effectively suppress beam-induced effect, thereby preserving the reliability of electrochemical observations, particularly those related to SEI and Li dendrites formation.

### Ex Situ Characterization

For ex situ imaging, the holder was first transferred to a glovebox, where the liquid electrolyte was removed. During disassembly of the cell the microchips were carefully retrieved to preserve the integrity of the membrane and the GC electrode. The chips were then gently cleaned with dimethyl carbonate (DMC) to eliminate residual organic species and mounted onto a Poseidon ex situ inspection holder. Finally, the holder was sealed in a plastic bag under an inert atmosphere and transferred to the microscope for high-resolution imaging and EDS analysis.

### Calibration of the Potential vs Li/Li^+^

All CV measurements in this study were performed using a Pt pseudoreference electrode. To accurately determine the electrochemical potentials versus Li/Li^+^, a calibration step was carried out using a solution of LiPF_6_ in a 3:7 (vol.) mixture of EMC and EC, containing 10 mmol/L ferrocene (Fe­(C_5_H_5_)_2_). CV scans were recorded to identify the redox peaks of the Fc/Fc^+^ couple relative to the Pt reference electrode (see Dachraoui et al.^5^ for experimental details). The half-wave potential, calculated as the average of the anodic and cathodic peak potentials, served as a reference value. Based on previous calibrations, this potential corresponds to approximately 3.24 V vs Li/Li^+^. This value was used to accurately estimate the vertex potentials applied during CV experiments investigating SEI formation and Li electrodeposition.

### Data Analysis and Image Preparation

To improve the contrast in specific regions, some figures were postprocessed. In Figure 3a the contrast of NP is highlighted with a temperature-based color code to enhance their visibility. Similarly, Figures 2d, and 4a,c use false color. Figure 4d features a series of FFT patterns with temperature-based color to enhance the visibility of the spots. Fiji (ImageJ) software was used to perform image adjustments.

## Supplementary Material













## Data Availability

The data that support the findings of this study are available from the corresponding author upon reasonable request.

## Notes

Details for the sample preparation, in situ liquid cell and methods for analyses, and additional figures as described in the text (PDF)
In situ ADF-STEM observation, showing the formation a layer of NPs at the edge of the GC electrode (AVI)
In situ ADF-STEM observation, showing NPs growing in size during charge process (AVI)
In situ ADF-STEM observation, showing some NPs growing in size during charge process (AVI)
In situ ADF-STEM observation, showing a Li whisker formed in the FEC-free electrolyte; it shows the dissolution of Li whiskers and the formation of dead Li (AVI)
In situ ADF-STEM observation, showing the dissolution of a Li whisker formed in the FEC-based electrolyte; it shows the complete dissolution of Li dendrites (AVI)
