# Supplementary material for: Enhanced Solid Electrolyte Interphase Layer in Li-Ion Batteries with Fluoroethylene Carbonate Additives Evidenced by Liquid-Phase Transmission Electron Microscopy
Source: ACS Nano. 2025 May 16;19(20):19213–24. doi: 10.1021/acsnano.5c01460 (PMC12120975; doi:10.1021/acsnano.5c01460)
Supplement: Supplementary file 1 [file nn5c01460_si_001.pdf]

Enhanced Solid Electrolyte Interphase Layer in Li-ion Battery with Fluoro-ethylene Carbonate Additive Evidenced by Liquid Phase Transmission Electron Microscopy

Walid Dachraoui[ab*], Ruben-Simon Kühnel[b], Nico Kummer[c], Corsin Battaglia[bdef], Rolf Erni[af*]

[a]Electron Microscopy Center, Empa—Swiss Federal Laboratories for Materials Science and Technology, Überlandstrasse 129, 8600 Dübendorf, Switzerland.
[b]Materials for Energy Conversion, Empa—Swiss Federal Laboratories for Materials Science and Technology, Überlandstrasse 129, 8600 Dübendorf, Switzerland.
[c]Transport at Nanoscale Interfaces Laboratory—Swiss Federal Laboratories for Materials Science and Technology, Überlandstrasse 129, 8600 Dübendorf, Switzerland
[d]Department of Information Technology and Electrical Engineering—ETH Zürich, Gloriastrasse 35, 8092 Zürich, Switzerland.
[e]Institute of Materials, School of Engineering – EPFL, Station 15, 1015 Lausanne, Switzerland
[f]Department of Materials – ETH Zürich, Wolfgang-Pauli-Strasse 10, 8049 Zürich, Switzerland.

Table of contents

Supplementary figures: Figures S1 to S11

Captions for movies: Movies S1 to S5

Decomposition of $LiPF_6$/EC/EMC

$LiPF_6 \longrightarrow LiF + PF_5$

$PF_5 + H_2O \longrightarrow POF_5 + 2HF$

$C_3H_4O_3 \longrightarrow C_2H_4 + CO_2$

EC $\longrightarrow$ CO_2 + ethylene (C_2H_4)

$C_4H_8O_3 \longrightarrow CO_2 + CH_3OCH_2CH_3$

EMC $\longrightarrow$ CO_2 + ethyl methyl ether ($CH_3OCH_2CH_3$)

Formation of the SEI layer involving $LiPF_6$ and EC/EMC

$2\ EC + Li^+ + e^- \longrightarrow (CH_2OCO_2Li)_2 + C_2H_4$

$2\ EMC + Li^+ + e^- \longrightarrow Li_2CO_3 + C_2H_5OCO_2Li + C_2H_6$

Formation of LiF

$FEC + Li^+ + e^- \longrightarrow LiF + C_2H_2O_2$

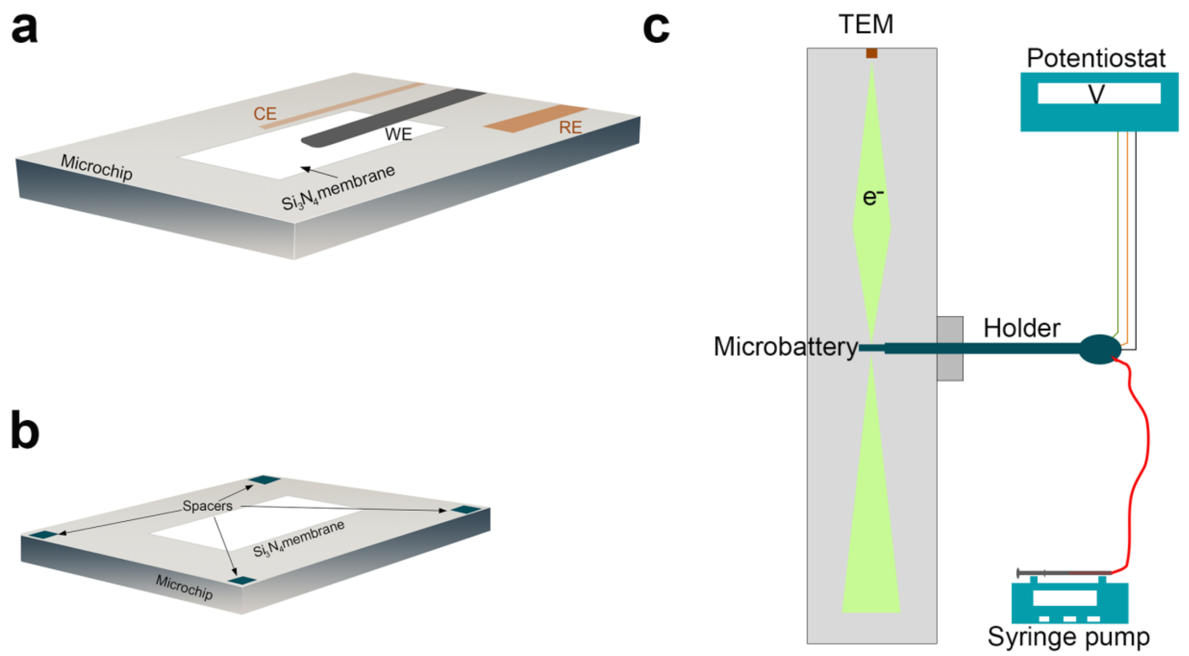

Figure S1. (a) Schematic illustration of the top microchip showing the working electrode (WE), reference electrode (RE), and counter electrode (CE). (b) Schematic illustration of the bottom microchip with spacers. (c) Schematic illustration of the ec-LC TEM setup: The in-situ liquid TEM holder is connected to a potentiostat via specialized external cables and to an external syringe pump via tubes, ensuring the injection of the electrolyte into the cell located in the TEM column.

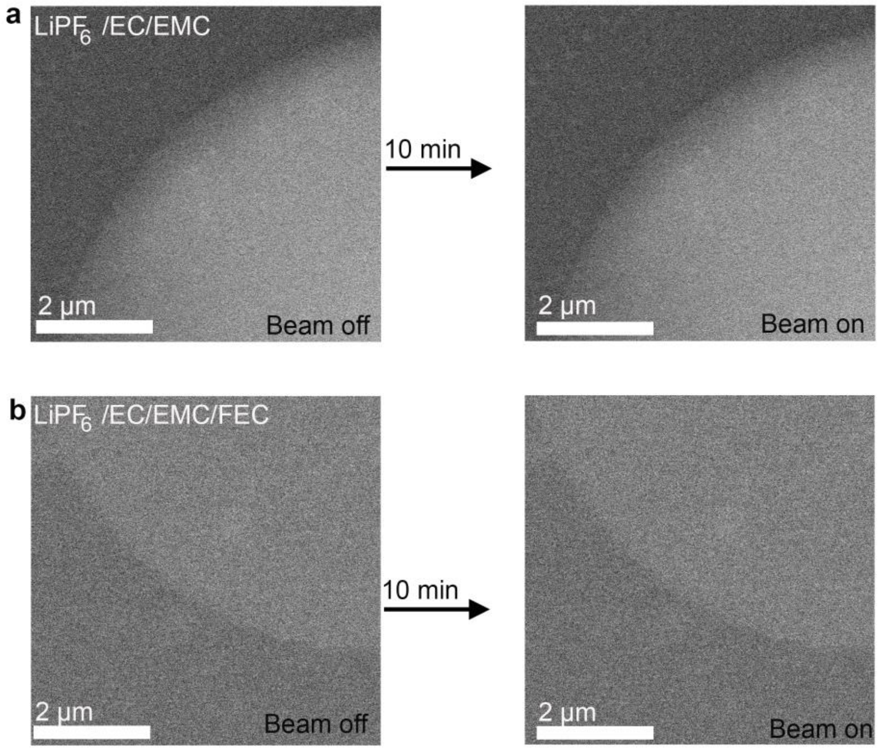

Figure S2. ADF-STEM images of the GC edge before and after 10 min of exposure to the electron beam. (a) Cell filled with $LiPF_6$/EC/EMC and (b) Cell filled with $LiPF_6$/EC/EMC with FEC.

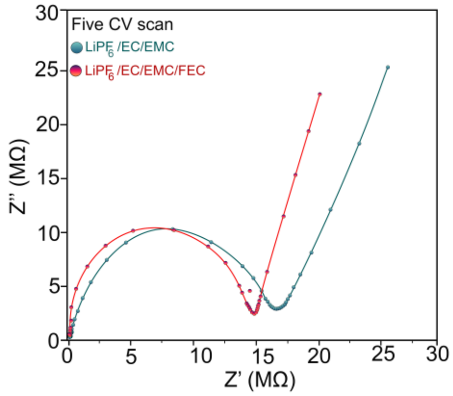

Figure S3. EIS curves after five cycles: pure electrolyte (green) and electrolyte with FEC (red).

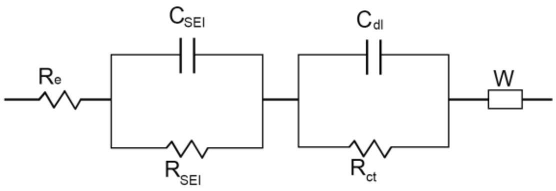

Figure S4: The equivalent circuit used to fit the EIS spectra in Figure 1. The fitting results are represented by lines in Figure 1d.

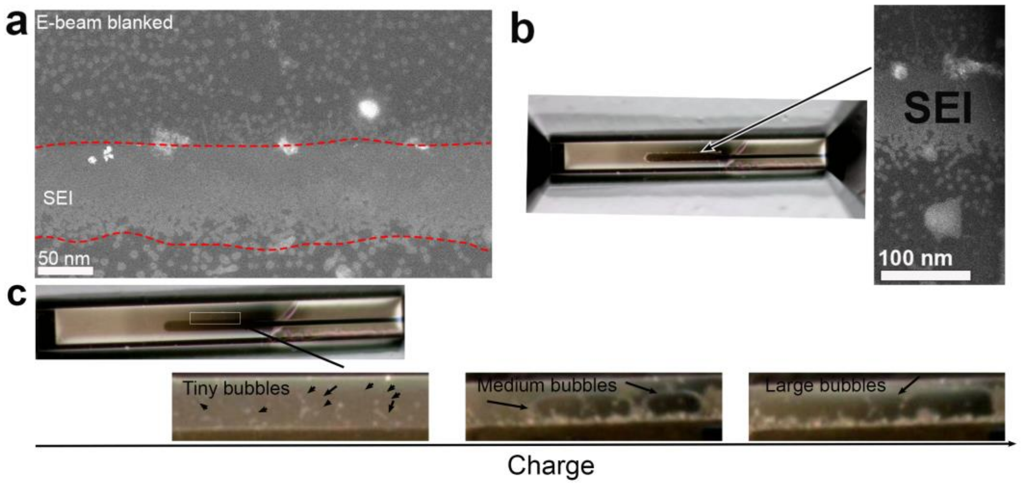

Figure S5. ADF-STEM images showing (a) the formation of gas bubbles during SEI formation and (b) the SEI formed after cycling with a blanked electron beam.

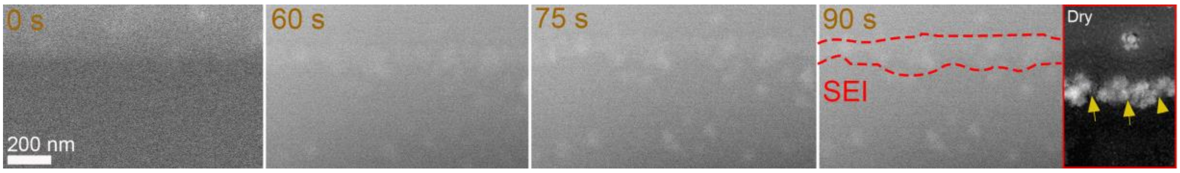

Figure S6. Time-lapse ADF-STEM images showing the growth of the SEI layer FEC-free electrolyte electrolyte.

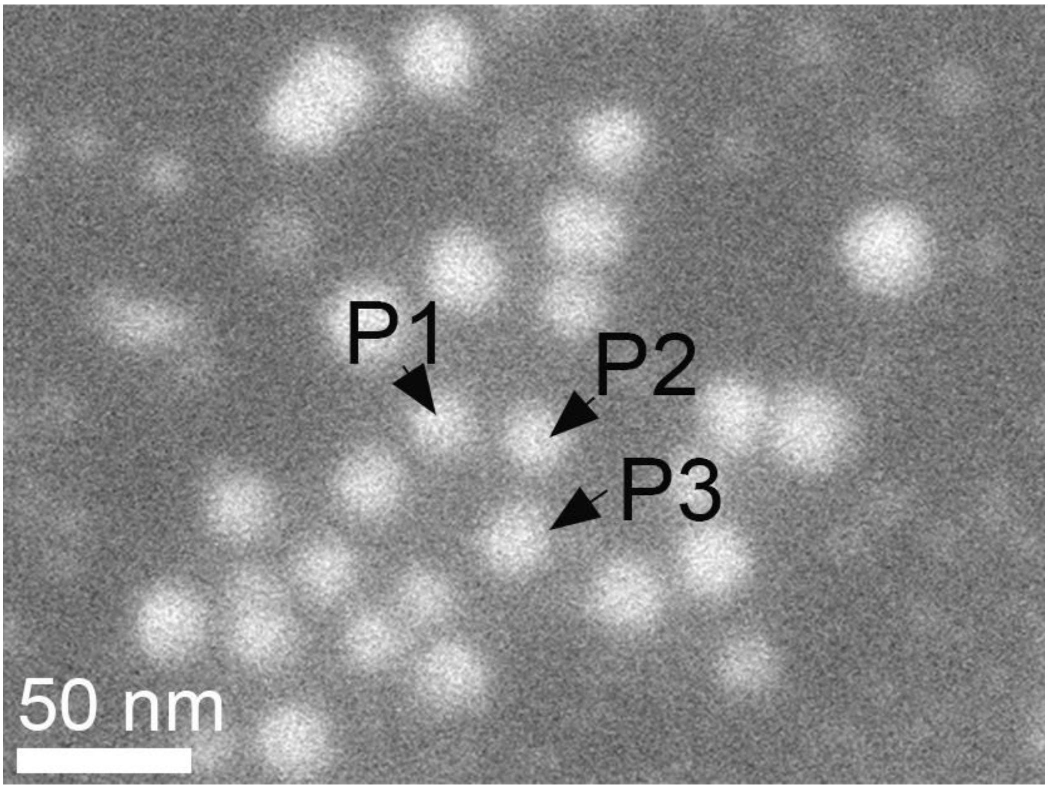

Figure S7. ADF-STEM image showing three typical NPs grown during the charge process.

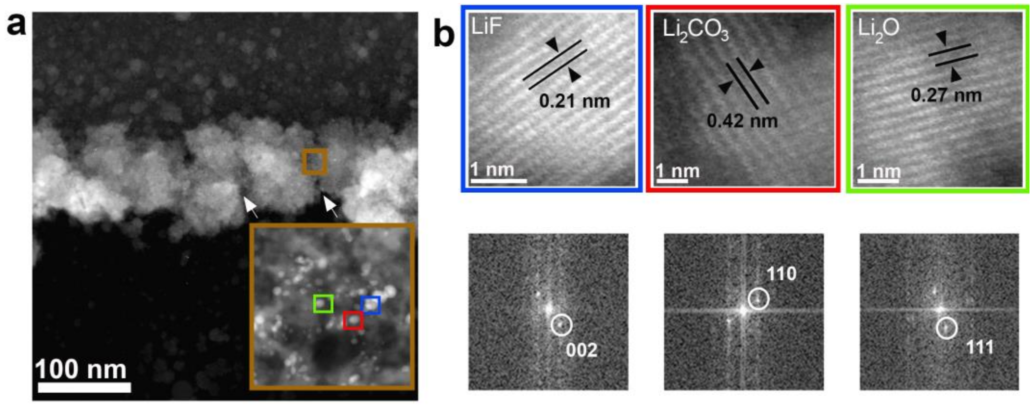

Figure S8. ADF-STEM images of the SEI grown in FEC-freeelectrolyte. (a) Overview of the formed SEI. (b) Three atomic-resolution ADF-STEM images with their corresponding FFT patterns from the regions highlighted with blue, red, and green squares in (a).

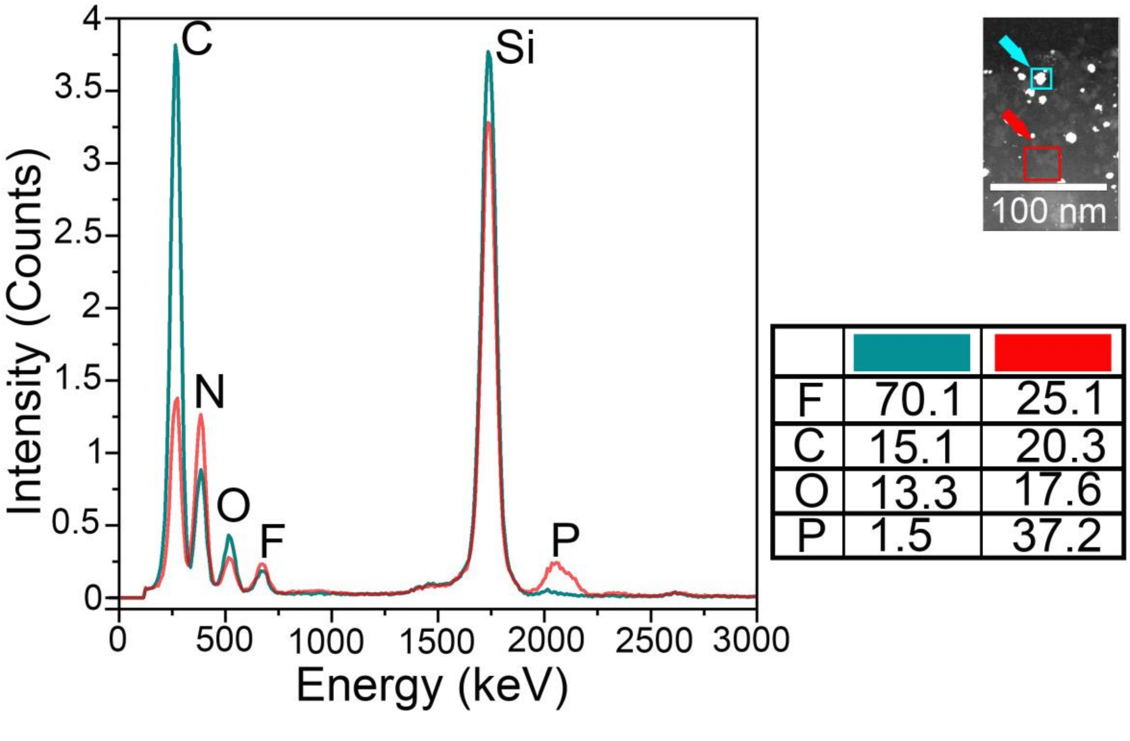

Figure S9. EDS spectra of different regions from the SEI layer, indicated by different colors.

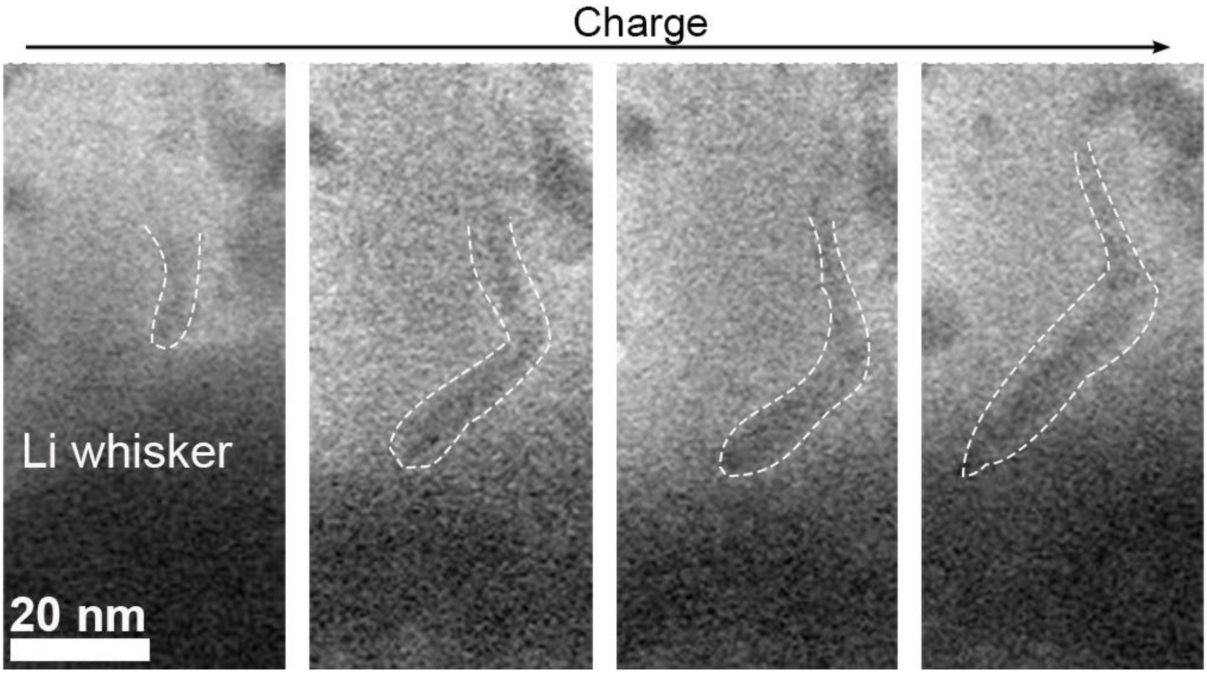

Figure S10. Time-lapse series of ADF-STEM images depicting the growth of a lithium whisker at the edge of the GC in FEC-free electrolyte.

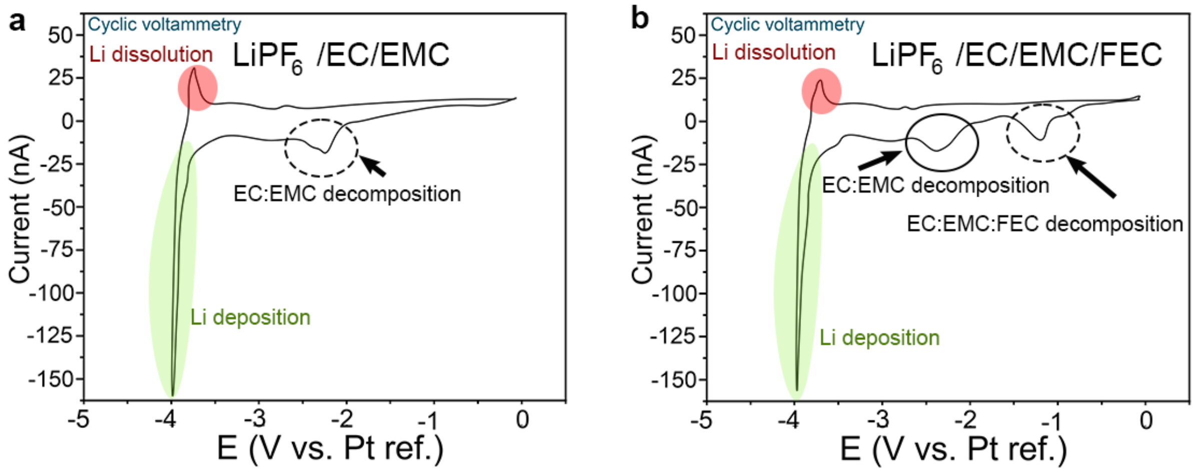

Figure S11. Two representative CV curves of the micro battery cycled in the electrolyte without additives (a) and with 10% FEC, displaying peaks for Li deposition and dissolution.

Captions for movies

Movie S1: SEI formation with FEC-based electrolyte decomposition product: It shows the formation a layer of NPs at the edge of the GC electrode. Scale bar 50 nm.

Movie S2: Growth of one NP: It shows one NP growing in size during the charge process. Scale bar 20 nm.

Movie S3: Growth of a patch of NPs: It shows some NPs growing in size during the charge process. Scale bar 20 nm.

Movie S4: Dissolution of a typical Li whisker formed in FEC-free electrolyte: It shows the partial dissolution of the Li whisker and the formation of dead Li. Scale bar 50 nm.

Movie S5: Dissolution of a typical Li dendrite formed in FEC-based electrolyte: It shows the complete dissolution of the Li dendrite. Scale bar 50 nm.
